# Nonlinear Dynamics Forecasting of Obstructive Sleep Apnea Onsets

**DOI:** 10.1371/journal.pone.0164406

**Published:** 2016-11-11

**Authors:** Trung Q. Le, Satish T. S. Bukkapatnam

**Affiliations:** 1 Department of Biomedical Engineering, International University-Vietnam National University, Ho Chi Minh, Vietnam; 2 Department of Biomedical Engineering, Texas A&M University, College Station, Texas, United States of America; 3 Center for Remote Health Technology, Texas A&M University, College Station, Texas, United States of America; Charité - Universitätsmedizin Berlin, GERMANY

## Abstract

Recent advances in sensor technologies and predictive analytics are fueling the growth in *point-of-care* (POC) therapies for obstructive sleep apnea (OSA) and other sleep disorders. The effectiveness of POC therapies can be enhanced by providing personalized and real-time prediction of OSA episode onsets. Previous attempts at OSA prediction are limited to capturing the nonlinear, nonstationary dynamics of the underlying physiological processes. This paper reports an investigation into heart rate dynamics aiming to predict in real time the onsets of OSA episode before the clinical symptoms appear. A prognosis method based on a nonparametric statistical Dirichlet-Process Mixture-Gaussian-Process (DPMG) model to estimate the transition from normal states to an anomalous (apnea) state is utilized to estimate the remaining time until the onset of an impending OSA episode. The approach was tested using three datasets including (1) 20 records from 14 OSA subjects in benchmark ECG apnea databases (Physionet.org), (2) records of 10 OSA patients from the University of Dublin OSA database and (3) records of eight subjects from previous work. Validation tests suggest that the model can be used to track the time until the onset of an OSA episode with the likelihood of correctly predicting apnea onset in 1 min to 5 mins ahead is 83.6 ± 9.3%, 80 ± 8.1%, 76.2 ± 13.3%, 66.9 ± 15.4%, and 61.1 ± 16.7%, respectively. The present prognosis approach can be integrated with wearable devices, enhancing proactive treatment of OSA and real-time wearable sensor-based of sleep disorders.

## 1. Introduction

Advancements in wearable sensors, flexible electronics, and “big data” predictive analytics have been encouraging the use of point-of-care (POC) technologies for obstructive sleep apnea (OSA) treatment. These include multiple variants of automatic positive airway pressure (APAP) machines, position-adjusting beds, and nerve stimulation devices. Such devices employ biomedical sensors to gather signals such as ECG, EMG, respiratory efforts, and SpO2 and to extract certain signal patterns for online adjustment of the device settings (e.g., air flow and body position). However, these adjustments tend to be reactive in that a control or intervention is initiated upon the detection of an OSA episode. We present an approach to predicting the time to OSA onset using nonlinear dynamics with the assumption that the underlying OSA-driven system equations are unknown and only the time series reflecting the evolution of the dynamics of the system are available. The timeliness and effectiveness of the intervention can be enhanced substantially if an impending OSA episode can be predicted before the clinical symptoms become evident. Such prediction information assists in adjustments of current OSA treatment devices by providing anticipatory information, before the onset of an OSA event.

**Fig 1:**
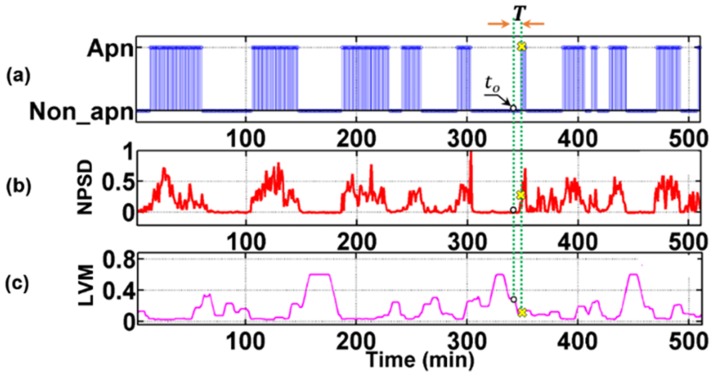
Time portraits of (a) minute-wise annotation of the presence (1) and absence (0) of OSA as marked offline by the physician based on polysomnography (PSG) records; (b) and (c) are variations of two significant quantifiers (NPSD and LVM) of HRV signals that are sensitive to the detection of OSA.

Although large streams of signals from multiple point-of-care sensors are becoming increasingly available, prediction (as opposed to detection) of OSA episodes from these signals—which is essential for proactive treatment—remains challenging. One of the major reasons is that the physiological processes underlying the measured physiological signals are highly nonlinear and nonstationary [1, 2]. Hence, the quantifiers extracted from these signals, also referred to as *features*, exhibit complex spatiotemporal patterns [3]. This challenge is exemplified by noting the evolution pattern of two features, namely the normalized band-limited power spectral density (NPSD) and the longest vertical recurrence length (LVM) extracted from the heart rate variability (HRV) signal of an apneic subject during sleep (see Fig 1). The amplitude of these features correlates strongly with the presence or absence of an OSA episode at a specified time. The key challenge in OSA prediction is predicting the onset of the sweeping change in the features and hence an OSA episode at the 350th min of sleep based on the observation of the preceding quiet behaviors. Such complex patterns are the result of the nonlinear and nonstationary dynamics of the underlying physiological processes and few of the current forecasting methods are known to predict these drastic transition behaviors.

Although the study of sleep dynamics and the transitions therein has evoked some interest [4, 5], an investigation into the dynamics of OSA inception is in its early stages. Bock and Gough utilized a nonlinear hidden layer neural network to predict the geometric invariants (e.g., largest Lyapunov exponent *λ*_*L*_ and correlation dimension *D*_*c*_) of the dynamic respiratory and cardiovascular systems in apneic sleeping patients [6]. Penzel et. al. suggested using exponential distributions to capture the duration of sleep stages (light, deep, and REM sleep combined) and a scale-free power law distribution of sleep stages to estimate the time to apnea [7]. Kim et al. posited that the duration of all sleep stages followed a modified exponential distribution and used a Markov process to model the durations between two consecutive sleep apnea events [8]. Waxman et al., utilized a neural network to predict wavelet transform-based features of six physiological signals obtained from a set of polysomnography studies to forecast up to 60 seconds in advance the occurrence of sleep apnea [9].

Nonetheless, very few (if any) research efforts have focused on quantifying the dynamics of the transitions from a normal to an abnormal (apneic and hypo-apneic) state during sleep for OSA prediction. Current OSA prediction methods have associated sleep stages with OSA transitions; however, the transitions in sleep stages are not necessarily related to the transition from a “normal” to an apneic state. Additionally, sleep dynamics prediction approaches are based on data gathered from a sample group of subjects to estimate the model parameters such as exponential decay, time scale, and the Markov transition matrix or to train the neural network for the prediction algorithms. Such approaches have limitations in capturing the inter-subject differences in dynamics necessary for personalized prognostics and treatment of OSA. Furthermore, sleep is a highly nonlinear and nonstationary physiological process in which the parameters describe the transition dynamics during the night. However, the current sleep dynamics methods assume the stationarity of the underlying dynamics, i.e., data gathered through an 8–10 hour sleep duration is used to estimate the model parameters. Such models are not able to provide effective predictions for out-of-sample data.

In this paper, we propose a computational method to characterize the complex relationships between the measured signals and the underlying OSA dynamics that enhances the effective prediction of OSA onset episodes. Specifically, we present (1) a method to characterize OSA dynamics and its state transitions based on the state space reconstructed from the measured signal features and (2) a personalized prognostic approach to provide in real time a statistical distribution of the time until the onset of an impending OSA episode based on a nonparametric statistical Dirichlet-Process Mixture-Gaussian-Process (DPMG). This work is a follow-up study continuing our previous work [10, 11] for the real-time prediction of time to sleep apnea onsets. The organization of the remainder of the paper is as follows: Section 2 describes the materials and method used; results are presented in Section 3, discussion and conclusions are provided in Sections 4 and 5, respectively.

## 2. Methods

### 2.1 Materials

Two datasets from the Physionet online database [12] have been used for this research. The first dataset is the collection of 20 records gathered from 14 OSA patients as part of the Apnea-ECG Database (http://physionet.org/physiobank/database/apnea-ecg/). The second dataset consists of 10 records from 10 subjects, who were selected based on the quality of the signals, from St. Vincent’s University Hospital/ University College Dublin (UCD Database) sleep apnea database (http://www.physionet.org/pn3/ucddb/). These online datasets are fully deidentified (anonymized) and can be used without further IRB approval. The OSA annotations are in minute-wise scored offline by experienced sleep physicians using polysomnography (PSG) signals. The PSG signals recorded were EEG, EOG, EMG, ECG, nasal airflow, ribcage movements, abdomen movements, oxygen saturation (finger pulse oximeter), snoring (tracheal microphone), and body position. All subjects have been clinically diagnosed with mild to severe OSA. The baseline characteristics of the patients from these two datasets are summarized in Table 1 with the specific details in the supporting information S1 and S2 Tables.

**Table 1:** Baseline characteristics of the patients from Apnea-ECG database and St. Vincent’s University Hospital/ University College Dublin Database (UCD database).

	Apnea-ECG Database	UCD Database
Measures (units)	Mean ± SD	Mean ± SD
No. of records/subjects	20/14	10/10
Age (yr)	52.1±6.5	48.4±8.18
BMI (kg/m^2^)	31.2±5.1	30.92±2.88
AHI (events/hr)	47.7±20.1	23.2±16.4
Study duration (min)	503.1±25.1	415.2±26.5
Apnea (min)	312.5±116.9	129.3±110.3
Non-apnea (min)	190.6±111.4	285.9±96.7

**BMI**: body mass index; **AHI**: apnea-hypoapnea index; **HI**: hypoapnea index; **AI**: apnea index

The third source of data is collected from eight voluntary male subjects using a wireless wearable device. The study obtained approval from the Oklahoma State University Institutional Review Board, application number EG125. The subjects signed a written consent form approved by the Institutional Review Board. The IRB approval and the continuations valid from Jan 14th, 2013 until Oct 1st, 2016 are included. Of the eight subjects participating the study, six are healthy and two are suspected of having sleep apnea. The subjects were trained to use the multisensory suite and the corresponding software. The suite is capable of synchronously gathering multiple heterogeneous signals, including VCG, ECG, snoring sounds, and respiration and wirelessly transmitting the data to a host computer for OSA prediction. In addition, a portable sleep monitoring device (from Zeo) with automated algorithms to distinguish between sleep and wakeful stages was used to record the sleep stages and rate the sleep quality using an average sleep score [13]. The detailed descriptions of the device and the experimental setup are in our previous work [10, 14].

**Fig 2:**
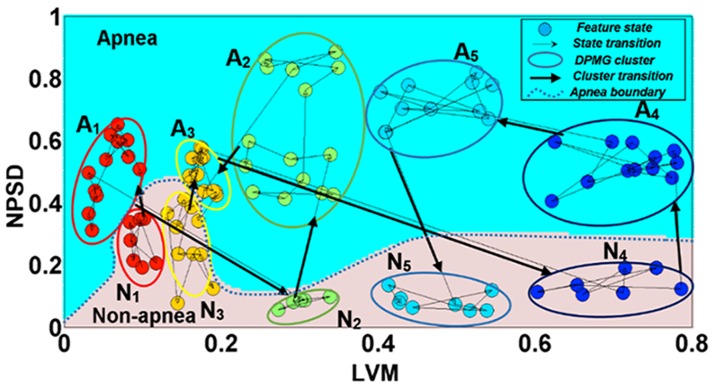
Evolution pathways of the features, namely NPSD and LVM, of heart rate variability signals for a representative subject during various sleep stages.

As stated above, the key idea is to monitor the transition pathways from a normal to an apneic state by tracking the evolution of the OSA dynamics underlying the measured heart rate variability signal. According to the HRV feature selection analysis [10], two features, namely the normalized power spectral density (NPSD) and the longest vertical length (LVM) of the recurrence plot, derived from the heart rate variability signal have been shown to be significant for the detection of OSA. Fig 2 shows a representative state transition pattern between apnea and non-apnea conditions in the NPSD and LVM feature space. The regions with dark (blue) background connote the feature values manifesting under non-apnea conditions and the light (gray) backgrounds under the apnea conditions. Each node in the figure denotes the *feature state* (i.e., NPSD and LVM values) realized at a particular time. The nodes’ color indicates the cluster (as determined by the DPMG model) to which the node is likely to belong. The **A**_**i**_ denotes apneic clusters i and **N**_**j**_ denotes nonapneic clusters j; the arrows among the nodes capture the evolution of the nodes over time.

It is observed in this figure that despite the evident clustering within the feature space, the dimension of the feature space is not high enough to capture the evolution patterns within and between clusters, as shown by excessive crisscrossing of the arrows. Therefore, the feature states should be embedded in a higher dimension in order to properly detangle and effectively quantify the evolution patterns and thereby provide an accurate prediction. The overall approach to addressing the challenges mentioned above consisted of (a) reconstructing a colored state space of suitably high dimension from the measured feature signals to track the evolution of the underlying processes, and (b) providing real-time prediction of OSA based on estimating the pathways and time distributions for OSA onset from any specified conditions in the colored state space. Additional details of the methods employed are summarized in the following sections.

### 2.2 State space reconstruction

We employed a multivariate delay reconstruction method [15] to embed the feature signals in a state space of suitable dimension that detangles the evolution patterns. A set of 14 features that quantify the spectral energy and nonlinear patterns (the so-called recurrence [16] patterns) of the heart rate variability signals is utilized for the OSA detection. Applying a statistical two-sample Kolmogorov Smirnov (KS) test (p<0.01), we found the two features denoted by NPSD and LVM to be the most sensitive in detecting OSA events [10]. Here, NPSD quantifies the distribution of the spectral density over the low-frequency band of 0.04–0.12 Hz. LVM, defined as the length of the recurrence plot’s longest vertical line, quantifies the extent of laminarity and the transitions to more complex regions (i.e., intermittency behavior) of the underlying dynamics. The recurrent plot is a visualization tool used for analyzing the recurrence characteristics of the phase space trajectory of the underlying dynamical system [16].

Next, the state space was partitioned into two sets corresponding to the feature values under apneic and non-apneic conditions. A support vector machine (SVM) classifier was employed to partition the state space into apnea and non-apnea areas. An SVM classifier is based on transforming the feature state space into an alternative space that can be partitioned into two subsets (apnea and non-apnea) by means of a single plane that is oriented as far away as possible from the feature values plausible for apneic and non-apneic conditions. Here we selected the radial basis function (RBF) as a transforming function based on its superior performance (mean sensitivity of 92.65% and specificity of 86.92%) compared to other classifiers in a k-fold cross-validation study.

Thereafter, we adopted a DPMG model [10] to cluster (and hence color code) the points in the state space as well as to predict the evolution of the signal features. In a DPMG model, the state space reconstructed from the signal features is assigned to various clusters according to a Dirichlet distribution. In each cluster, a local Gaussian process (LGP) prediction model has been utilized to forecast the evolution of the features from each point in the sampled state space. An LGP prediction provides not only a point estimator but also the distribution of the future state based on the prior estimation of the characteristics of the local clusters that the current observation is assigned to. In this present paper, we characterized the evolution pattern of the predicted features in the state space to quantitatively forecast the time to the impending OSA onsets.

#### 2.2.1 Real-time OSA event prognosis

The real-time prognosis method is based on concatenating the evolution patterns gleaned from DPMG forecasts over the colored state space. First, the state space is discretized into 20 blocks along every dimension (quantization) and transformed into a lower dimensional space to enhance the computational speed. Every quantized block *i* serves as a node, *v* ∈ *V*, of a random directed graph, *G*(*V*, *P*), which is connected by directed arcs (arrows) whose weights are the probabilities, *P*, of the evolution between the two nodes (*i* through *j*) in the state space. Tracking the evolution pathway, characterized as a series of transitions, through the nodes of the network embedded in high dimensional state space tends to be unwieldy. We have therefore utilized a Laplacian-based projection [17] to reduce the dimensionality of the quantized state space. In this projection technique, the nodes with the higher probability of belonging to an evolution from a non-apnea state to an apnea state are relocated closer. By positioning all nodes with the same evolutions together, evolution pathways from a non-apnea state to an apnea state in the discretized state space are untangled from the cross-over state transitions in the original 10-dimensional state space specified by the false nearest neighbors test [18] of the underlying OSA dynamics. Hence, Laplacian feature projection enhances the characterization and quantifying of the OSA states’ evolutions necessary for realizing the prognosis step.

The real-time prognosis method—concerned with estimating the distribution of time until an apnea onset occurs—is based on concatenating the stochastic evolution patterns of the transitions from a non-apneic to an apneic state in the developed color state space. Since the future state of any node in the discretized network depends on its time-shifted components, the transition probability among the nodes constitutes a Markov chain [19]. The Markov property of the transition among the nodes in the discretized state space network allows an efficient means of estimating the *P*_*ij*_. Here, we first employed a one-step prediction of the DPMG model to determine the probability of transition *P*_*ij*_ from every block to every other block. Next, the evolution path is formed by concatenating the transitions from one non-apnea block to its predicted blocks in the discretized state space. The concatenation process is repeated until the predicted block of the path is an apnea block. The distribution of time until an apnea onset from a specific non-apnea block *i* in the state space is estimated from the number of transitions in all possible evolution paths from block *i* to the first apneic state block as $Pr\left[T=i{|\text{x}}_{*}\right]=\left(1-P_{k}\right)\sum_{l_{1}=1}^{n}\ldots \sum_{l_{i-2}=1}^{n}\sum_{l_{i-1}1}^{n}A_{l_{1}}^{k}\ldots A_{l_{i-2}}^{l_{i-3}}A_{l_{i-1}}^{l_{i-2}}\left(1-P_{l_{1}}\right)\ldots \left(1-P_{l_{i-2}}\right)P_{l_{i-1}}$. A detailed mathematical description of the concatenation process is provided in the S1 File.

## 3. Results

### 3.1 Characterization of OSA state space

**Fig 3:**
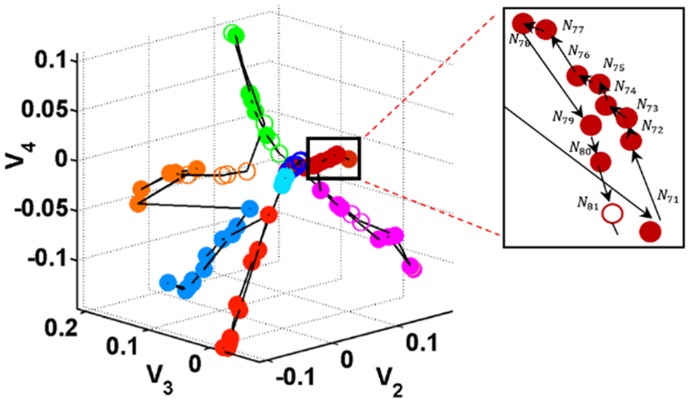
Distribution of the feature states in the Laplacian-based coordinate system (V_2_, V_3_ and V_4_) of a representative record.

Based on the false nearest neighbors test [18], our investigation consistently suggests that the dynamics underlying the measured heart rate variability signal have a ten-dimensional state space. Such a high dimension is not suitable for tracking the evolution pattern of the feature transitions. The Laplacian eigenvector coordinate system has been employed to accommodate such an evolution of the feature states. Fig 3 illustrates the evolution of the features in 3-D Laplacian-based coordinates over time of a representative record. Each node connotes the feature state realized at a particular time. The color of the node indicates the cluster (as determined by the DPMG model) to which the node is likely to belong, with shape showing the non-apneic (open circle) and apneic (closed circle) conditions and the connectors among the nodes annotating the features’ evolution over time. Although the feature values of the M-dimensional (M≈10) state space are projected into a lower dimensional space (i.e., 3-dimensional Laplacian-based coordinates), its variations are preserved and the separation of the features into different clusters is enhanced. This is shown by the disjoined clusters in different colors and the dense nodes in the same cluster. Furthermore, in each local cluster, states with high probabilities, shown as adjacent points in the evolution pathways, are more likely to be relocated near each other as shown in the zoomed subplot. The transitions among nodes in the state space can be effectively characterized on a global (between clusters) or local (within cluster) scale.

### 3.2 Prognostics performance

#### 3.2.1 Prediction of time to OSA onsets

**Fig 4:**
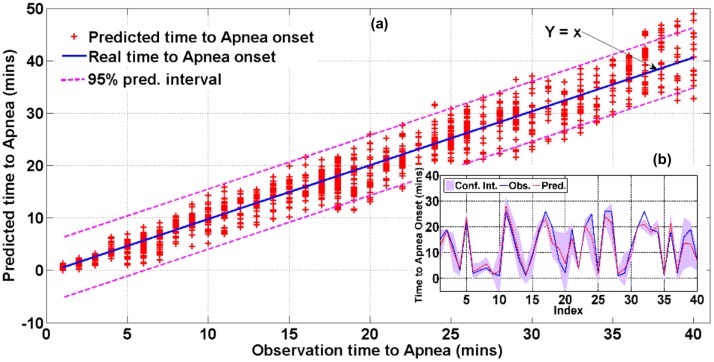
Scatter plot of prediction versus real observation of time to apnea (a) and run plot showing the predictions of the expected time to sleep apnea onset in a representative patient (b).

The accuracy of the prediction model denoted by the deviation of the prediction from the ground truth is evaluated. All predicted times to apnea and the real observations (a) and the expected times to sleep apnea onset over 40 random points selected from the testing data of a representative patient (b) are reported in Fig 4. The observations of the time to apnea onset (ground truth) are indicated in blue, the expected values from the distribution of time to failure are in red, and the 95% confidence intervals of the prediction values are within the purple strips. In Fig 4a, the prediction error increases as the prediction horizon increases from 1 to 40 min ahead; however, 95% of the predictions are still within the 95% confidence interval. Fig 4b shows the prediction accuracy in a representative patient with the times the predictions are made selected randomly from the testing data. Accordingly, the average determination coefficient (*R*^2^) between the expected time to apnea onset from the estimated distribution of time to OSA and the real observations from the testing data are reported as 0.8, and 91% of the observations are within the 95% confidence interval of the prediction.

**Fig 5:**
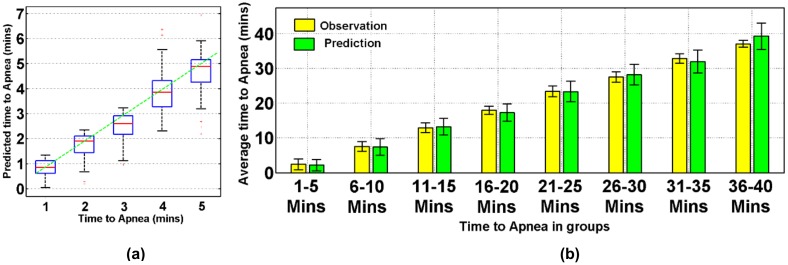
(a) Prediction performance using randomly selected data from the testing data set for a 1–5 min-ahead prediction with a prediction horizon increment of 1 min and (b) a 1–40 min-ahead prediction with a prediction horizon increment of 5 min.

**Fig 6:**
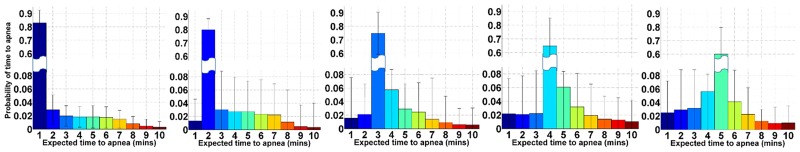
Distribution of the expected time to apnea onset estimated from f_T_(t|x_*_) for 1–5 min ahead from left to right.

The precision characterizing the variation of the predictions along with the equivalent ground truths is investigated. The distribution of the estimated time to apnea onset and the prediction errors are shown in Fig 5(a) with1-5 min-ahead predictions for a prediction horizon increment of 1 min and in Fig 5(b) with 1–40 min-ahead predictions for a prediction horizon increment of 5 min. In both figures, the horizontal axis shows the actual time to apnea onset and the vertical axis shows the expected time to apnea onset estimated from the distribution of time to apnea onset. For Fig 5, the groups in the results are retained using randomly selected data from the testing data set. Note that the observation values are within the upper and lower quartiles of the predicted time to apnea onset in Fig 5(a) and that the prediction intervals cover the expected observation values in Fig 5(b) Also, note from Fig 5(a) that the prediction errors decrease with the decrease in prediction horizons. We also see this trend in Fig 6(b) in terms of absolute residuals, when the prediction horizon decreases to a 1–5 min-ahead prediction.

#### 3.2.2 Risk indicators for OSA onset

The prognostic performance is further investigated by analyzing the variations in the estimated risk indicators for apnea episode occurrence. The estimated risk indicators specify the likelihood that the apnea event will happen within a specific time. Fig 6 shows the distribution of the risk indicators at 1–5 min preceding sleep apnea onset. For example, the risk indicator at 3-min to the OSA onset equals the probability of the distribution of time to failure evaluated at *t* = 3. The average estimate of risk indicators at 1 to 5 min to apnea onset are 83.6±9.3%, 80±8.1%, 76.2±13.3%, 66.9±15.4%, and 61.1±16.7% respectively. Note that the risk indicator is relative (84% ±5.3%). The estimated risk indicators at 1–3 min until apnea onset are all higher than 75%. Such a high prognosis performance can provide reliable supportive information for preventive treatment before the actual apnea event happens.

#### 3.2.3 Pre-clinical preliminary results

**Fig 7:**
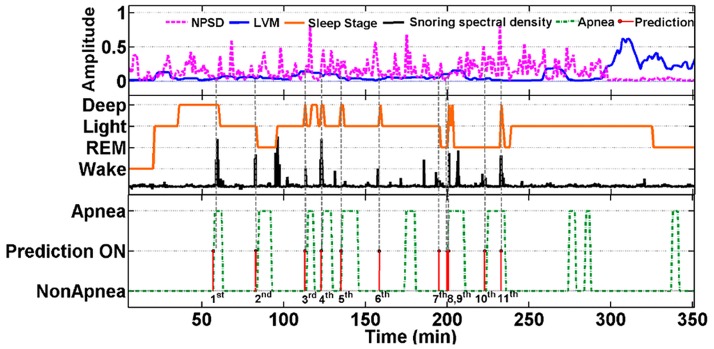
The waveforms of NPSD, LVM, sleep stage pattern, spectral density of snoring sound, OSA annotation and one-minute ahead prediction of sleep apnea in a subject.

In addition to evaluating the prediction performance using data from the online databases, we also evaluated our model using data collected by a patented wireless wearable device [20] from eight subjects. Fig 7 shows a representative 350-min long signal segment collected from a subject who donned the multisensory suite to sleep for 434 minutes. This subject suspects that he suffers from sleep apnea and shows several signs of sleep apnea including loud snoring and disturbed sleep. The signals in Fig 7 include synchronously gathered NPSD (purple), LVM (blue), sleep stage (orange), and snoring sound (black), sleep apnea annotations (green) denoted by the SVM classifier, and offline one-minute-ahead sleep apnea predictions (red) estimated from the distribution of time to apnea onset. All evaluations are made after the subject falls asleep, as indicated by the transition from an awake to a light sleep stage (around the 20^th^ min). Note that no apneic episode was predicted during the 25 min long deep sleep, or during the stable light sleep stage in the 200–300 min range. More pertinently, the first apnea event predicted at the 60 min mark precedes the snoring/breathing episode and the transition from a deep to a light sleep stage. Also, the 2^nd^, 3^rd^, 4^th^, 5^th^, 6^th^, 7^th^, 8^th^, 9^th^ and 11^th^ apnea events are predicted near the transitions from a deep sleep to a light or REM sleep stage at the onset of apnea annotation from the offline apnea classification. One apparent false positive apnea prediction (10^th^) occurred at the 225^th^ min mark in the REM sleep stage. Additional training in estimating the parameters of the classification and prediction models using the subject’s longitudinal data might further improve the prediction sensitivity and specificity.

## 4. Discussion

The proposed OSA prediction method is based on forecasting the elevated sympathetic tones and the nonlinear transition changes characterized by the power spectral density and recurrence features of the HRV signals. The nonlinear dynamics of the heart rate variability originate from the variations in ion channels [21] and the neural impulses [22] controlled by the sympathetic division (SYD) and the parasympathetic division (PYD) during OSA episodes. Several studies have investigated the spectral components of the low frequency (0.04–0.1 Hz) of the HRV and concluded that the power spectral density of the low frequency is highly associated with the elevated sympathetic tones during the OSA period [23]. In addition, the longest vertical length feature of the recurrence quantification analysis quantifies the extent of the laminar states and the transitions to more complex regions (i.e., intermittency behavior). Previous works have demonstrated that the longest vertical lengths are significant features in characterizing transitions in the HRV dynamic regulation system. Physiologically, our prediction model utilizes a nonparametric statistical DPMG model to capture the variations in the HRV features associated with pending OSA episodes.

To further support the hypothesis that sleep apnea is a complex process whose pathophysiological dynamics are intertwined among different physiological dynamic systems, several limitations of the model need to be addressed. The nonlinear dynamic state space was reconstructed based on features from heart rate variability only. Features from physiological processes, such as respiratory (impaired upper airway and lung structure and unstable ventilatory control) [24] and neurological (deficient neuromechanical and neurochemical control mechanism) [25, 26] systems have not been included in the model. Hence to have a better understanding of the coupled dynamics, the different causal mechanisms of sleep apnea, and the correlations among these mechanisms need to be considered. Furthermore, stochastic model components need to be introduced to account for the various exogenous (stress, food intake, sleep deprivation, sensorial stimulation) [27] and endogenous (neurotransmitter, peptide, and hormone) [28] random factors that regulate the sleep-wake cycles. Such modifications may provide a better understanding of sleep stages and sleep apnea mechanisms.

## 5. Conclusions

We have introduced a color-coded state space representation for characterizing the dynamics underlying the OSA process and a novel method for estimating the remaining time until the onset of the pending OSA episode based on a nonparametric statistical prediction model (i.e., DPMG). The approach was tested using 30 data records from 24 subjects in benchmark apnea databases (Physionet.org), as well as from 8 subjects wearing a sensor unit during sleep. Investigation shows that the proposed color-coded state presentation provides an effective characterization tool for tracking the evolution patterns from a non-apneic to an apneic state in the state space of the underlying OSA dynamic process. The risk indicators derived from the time to OSA onset distribution are 83.6 ± 9.3%, 80.2 ± 8.1%, and 76.2 ± 13.3% at 1 to 3 min to apnea onset, respectively. Such early predictions with reliable risk indicators can be used to support preventive treatment and mitigate the consequences of acute disorders.

## Supporting Information

S1 File Derivation of the distribution of time to apnea. (DOCX)

S1 Table Diagnostic information of the OSA patients from Apnea-ECG Database- Physionet with the colored records show the same patient collected over multiple nights. (DOCX)

S2 Table Diagnostic information of the OSA patients from St. Vincent’s University Hospital/ University College Dublin. (DOCX)
