# Correction: Nonlinear Dynamics Forecasting of Obstructive Sleep Apnea Onsets

**DOI:** 10.1371/journal.pone.0183422

**Published:** 2017-08-10

**Authors:** Trung Q. Le, Satish T. S. Bukkapatnam

There is an error in affiliation 2 for author Satish T. S. Bukkapatnam. Affiliation 2 should be: Department of Industrial and Systems Engineering, Texas A&M University, College Station, Texas, United States of America.

A sentence and a reference are missing from the second paragraph of the Introduction. The third sentence of the second paragraph in the Introduction section should read: Specifically these signals exhibit dynamic intermittencies and other transient behaviors (Cheng, Changqing, et al., 2015).

The reference is: Cheng, Changqing, et al. "Time series forecasting for nonlinear and non-stationary processes: a review and comparative study." *IIE Transactions* 47.10 (2015): 1053–1071.

There are errors in the second sentence of the Discussion. The correct sentence is: Nonlinear dynamics of HRV signals originate from the variations in ion channels [21] and the neural impulses [22] controlled by the sympathetic division (SYD) and the parasympathetic division (PYD) during OSA episodes.
