# Supplementary material for: Nonlinear Dynamics Forecasting of Obstructive Sleep Apnea Onsets
Source: PLoS One. 2016 Nov 11;11(11):e0164406. doi: 10.1371/journal.pone.0164406 (PMC5105938; doi:10.1371/journal.pone.0164406)
Supplement: S1 File — (DOCX) [file pone.0164406.s001.docx]

*S1 File. Derivation of the distribution of time to apnea*

*In the probability space* $(\Omega\mathcal{,F,}P)$ *where* $\mathcal{F}$ *= {1, 0}, 1 represents for apnea event and 0 for non-apnea event, the probability distribution function of time to apnea onset* $T$ *is defined as* $f_{T}\left( t | x_{*}=k \right)=Pr[X_{t}\left( W \right)=1|\wedge(X_{1}(W)=0, \ldots,X_{t-1}\left( W \right)=0)$*],*$t =1,\ldots.n$*.* $X_{T}\left( W \right)$ *is defined as {*$\forall W, W\in\Psi, X\left( W \right)\mathcal{\in F}$*} where* $\Psi\in L^{3}$*. At a specific non-apnea block* $x_{*}$*in the discretized state space, the probability of time to apnea onset* $T=i$*denoted as*$Pr\left[ T=i | x_{*} \right]=\left( 1-P_{k} \right)\sum_{l_{1}=1}^{n} \ldots\sum_{l_{i-2}=1}^{n} \sum_{l_{i-1}1}^{n} A_{l_{1}}^{k}\ldots A_{l_{i-2}}^{l_{i-3}}A_{l_{i-1}}^{l_{i-2}}\left( 1-P_{l_{1}} \right)\ldots\left( 1-P_{l_{i-2}} \right)P_{l_{i-1}},$*equal to the probability that non-apnea block* $x_{*}$ *evolves over* $i-1$*non-apnea blocks and stops in an apnea block at step* $i^{th} . \boldsymbol{P}={[P}_{1}\ldots.P_{n}]$ *is the estimated probability of* $n$ *points in state space (* $L_{1}, L_{2},$*and* $L_{3}$*) to sleep apnea in 1 step and* $\boldsymbol{A}^{i}=[A_{1}^{i},\ldots.A_{n}^{i}]$ *is* *transition* *coefficients to all states from state* $i$*. Here, the evolution patterns over* $i-1$*non-apnea blocks from block* $x_{*}$*are concatenated from the transitions of* $x_{*}$ *through i-1 possible blocks in the quantized state space. The probabilities of being in an apnea block one step ahead of every non-apnea block in the discretized state space is updated using one-step-ahead predictions from the DPMG model.*
