# Supplementary material for: Nonlinear Dynamics Forecasting of Obstructive Sleep Apnea Onsets
Source: PLoS One. 2016 Nov 11;11(11):e0164406. doi: 10.1371/journal.pone.0164406 (PMC5105938; doi:10.1371/journal.pone.0164406)
Supplement: S1 Table — (DOCX) [file pone.0164406.s002.docx]

**Supporting Information**

**S1 Table. Diagnostic information of the OSA patients from Apnea-ECG Database- Physionet with the colored records show the same patient collected over multiple nights**

| **Record** | **Patient ID** | **Sleep Duration (min)** | **Apnea**  **(min)** | **AI** | **HI** | **AHI** | **Age** | **Height**  **(cm)** | **Weight**  **(kg)** | **BMI** |
| --- | --- | --- | --- | --- | --- | --- | --- | --- | --- | --- |
| a01 | p1 | 490 | 470 | 13 | 57 | 70 | 51 | 175 | 102 | 33.31 |
| a02 | p2 | 529 | 420 | 57 | 12 | 70 | 38 | 180 | 120 | 37.04 |
| a03 | p3 | 520 | 246 | 38 | 1 | 39 | 54 | 168 | 80 | 28.34 |
| a04 | p4 | 493 | 453 | 73 | 4 | 77 | 52 | 173 | 121 | 40.43 |
| a05 | p5 | 455 | 276 | 35 | 6 | 41 | 58 | 176 | 78 | 25.18 |
| a06 | p6 | 511 | 206 | 17 | 8 | 25 | 63 | 179 | 104 | 32.46 |
| a07 | p7 | 512 | 322 | 46 | 17 | 63 | 44 | 177 | 105 | 33.52 |
| a08 | p8 | 502 | 189 | 32 | 10 | 42 | 51 | 179 | 88 | 27.46 |
| a09 | p9 | 496 | 381 | 23 | 9 | 32 | 52 | 178 | 82 | 25.88 |
| a10 | p5 | 518 | 100 | 11 | 10 | 21 | 58 | 176 | 78 | 25.18 |
| a11 | p10 | 467 | 222 | 11 | 3 | 14 | 58 | 168 | 103 | 36.49 |
| a12 | p4 | 578 | 534 | 70 | 10 | 80 | 52 | 173 | 121 | 40.43 |
| a13 | p8 | 496 | 244 | 32 | 10 | 42 | 51 | 179 | 88 | 27.46 |
| a14 | p1 | 510 | 383 | 17 | 37 | 55 | 51 | 175 | 102 | 33.31 |
| a15 | p11 | 511 | 368 | 46 | 6 | 52 | 60 | 176 | 113 | 36.48 |
| a16 | p7 | 483 | 320 | 17 | 24 | 41 | 44 | 177 | 105 | 33.52 |
| a17 | p12 | 486 | 158 | 21 | 12 | 33 | 40 | 179 | 96 | 29.96 |
| a18 | p9 | 490 | 438 | 76 | 7 | 82 | 52 | 178 | 82 | 25.88 |
| a19 | p13 | 503 | 205 | 34 | 0 | 34 | 55 | 178 | 90 | 28.41 |
| a20 | p14 | 511 | 315 | 35 | 6 | 41 | 58 | 176 | 78 | 25.18 |
| **Average** | | 503.05 | 312.50 | 35.20 | 12.47 | 47.67 | 52.10 | 176.00 | 96.80 | 31.30 |
| **StdDev** | | 25.09 | 116.90 | 20.62 | 13.45 | 20.14 | 6.54 | 3.36 | 14.93 | 5.09 |

**S2 Table. Diagnostic information of the OSA patients from St. Vincent’s University Hospital/ University College Dublin.**

| No | Patient ID | Study Duration (Min) | Apnea  (Min) | AI | HI | AHI | Age | Height  (cm) | Weight  (kg) | BMI |
| --- | --- | --- | --- | --- | --- | --- | --- | --- | --- | --- |
| 1 | UCDDB002 | 372 | 110 | 2 | 21 | 23 | 54 | 172 | 100.3 | 33.9 |
| 2 | UCDDB003 | 438 | 321 | 5 | 46 | 51 | 48 | 179 | 102 | 31.8 |
| 3 | UCDDB012 | 432 | 136 | 6 | 19 | 25 | 51 | 179 | 97.5 | 30.4 |
| 4 | UCDDB013 | 408 | 61 | 2 | 14 | 16 | 62 | 153 | 80 | 34.2 |
| 5 | UCDDB018 | 408 | 9 | 0 | 2 | 2 | 35 | 171 | 77 | 26.3 |
| 6 | UCDDB020 | 378 | 64 | 2 | 13 | 15 | 52 | 179 | 108.8 | 34 |
| 7 | UCDDB022 | 396 | 27 | 3 | 4 | 7 | 34 | 166 | 80.7 | 29.3 |
| 8 | UCDDB024 | 456 | 139 | 4 | 20 | 24 | 54 | 172 | 99.9 | 33.8 |
| 9 | UCDDB026 | 420 | 78 | 3 | 11 | 14 | 49 | 175 | 84 | 27.4 |
| 10 | UCDDB027 | 444 | 348 | 18 | 37 | 55 | 45 | 182 | 93 | 28.1 |

*S1 File. Derivation of the distribution of time to apnea*

*In the probability space* $(\Omega\mathcal{,F,}P)$ *where* $\mathcal{F}$ *= {1, 0}, 1 represents for apnea event and 0 for non-apnea event, the probability distribution function of time to apnea onset* $T$ *is defined as* $f_{T}\left( t | x_{*}=k \right)=Pr[X_{t}\left( W \right)=1|\wedge(X_{1}(W)=0, \ldots,X_{t-1}\left( W \right)=0)$*],*$t =1,\ldots.n$*.* $X_{T}\left( W \right)$ *is defined as {*$\forall W, W\in\Psi, X\left( W \right)\mathcal{\in F}$*} where* $\Psi\in L^{3}$*. At a specific non-apnea block* $x_{*}$*in the discretized state space, the probability of time to apnea onset* $T=i$*denoted as*$Pr\left[ T=i | x_{*} \right]=\left( 1-P_{k} \right)\sum_{l_{1}=1}^{n} \ldots\sum_{l_{i-2}=1}^{n} \sum_{l_{i-1}1}^{n} A_{l_{1}}^{k}\ldots A_{l_{i-2}}^{l_{i-3}}A_{l_{i-1}}^{l_{i-2}}\left( 1-P_{l_{1}} \right)\ldots\left( 1-P_{l_{i-2}} \right)P_{l_{i-1}},$*equal to the probability that non-apnea block* $x_{*}$ *evolves over* $i-1$*non-apnea blocks and stops in an apnea block at step* $i^{th} . \boldsymbol{P}={[P}_{1}\ldots.P_{n}]$ *is the estimated probability of* $n$ *points in state space (* $L_{1}, L_{2},$*and* $L_{3}$*) to sleep apnea in 1 step and* $\boldsymbol{A}^{i}=[A_{1}^{i},\ldots.A_{n}^{i}]$ *is* *transition* *coefficients to all states from state* $i$*. Here, the evolution patterns over* $i-1$*non-apnea blocks from block* $x_{*}$*are concatenated from the transitions of* $x_{*}$ *through i-1 possible blocks in the quantized state space. The probabilities of being in an apnea block one step ahead of every non-apnea block in the discretized state space is updated using one-step-ahead predictions from the DPMG model.*
