# Supplementary material for: Nonlinear Dynamics Forecasting of Obstructive Sleep Apnea Onsets
Source: PLoS One. 2016 Nov 11;11(11):e0164406. doi: 10.1371/journal.pone.0164406 (PMC5105938; doi:10.1371/journal.pone.0164406)
Supplement: S2 Table — (DOCX) [file pone.0164406.s003.docx]

**S2 Table. Diagnostic information of the OSA patients from St. Vincent’s University Hospital/ University College Dublin.**

| No | Patient ID | Study Duration (Min) | Apnea  (Min) | AI | HI | AHI | Age | Height  (cm) | Weight  (kg) | BMI |
| --- | --- | --- | --- | --- | --- | --- | --- | --- | --- | --- |
| 1 | UCDDB002 | 372 | 110 | 2 | 21 | 23 | 54 | 172 | 100.3 | 33.9 |
| 2 | UCDDB003 | 438 | 321 | 5 | 46 | 51 | 48 | 179 | 102 | 31.8 |
| 3 | UCDDB012 | 432 | 136 | 6 | 19 | 25 | 51 | 179 | 97.5 | 30.4 |
| 4 | UCDDB013 | 408 | 61 | 2 | 14 | 16 | 62 | 153 | 80 | 34.2 |
| 5 | UCDDB018 | 408 | 9 | 0 | 2 | 2 | 35 | 171 | 77 | 26.3 |
| 6 | UCDDB020 | 378 | 64 | 2 | 13 | 15 | 52 | 179 | 108.8 | 34 |
| 7 | UCDDB022 | 396 | 27 | 3 | 4 | 7 | 34 | 166 | 80.7 | 29.3 |
| 8 | UCDDB024 | 456 | 139 | 4 | 20 | 24 | 54 | 172 | 99.9 | 33.8 |
| 9 | UCDDB026 | 420 | 78 | 3 | 11 | 14 | 49 | 175 | 84 | 27.4 |
| 10 | UCDDB027 | 444 | 348 | 18 | 37 | 55 | 45 | 182 | 93 | 28.1 |
